# MiR-218-5p targets LHFPL3 to regulate proliferation, migration, and epithelial–mesenchymal transitions of human glioma cells

**DOI:** 10.1042/BSR20180879

**Published:** 2019-03-01

**Authors:** Zhixiao Li, Rongjun Qian, Jiadong Zhang, Xiwen Shi

**Affiliations:** Department of Neurosurgery, The People’s Hospital of Zhengzhou University, Zhengzhou, Henan 450003, People’s Republic of China

**Keywords:** EMT, glioblastoma, has-miR-218-5p, Lipoma HMGIC fusion partner-like 3

## Abstract

Glioblastoma (GBM) is a main subtype of high-grade gliomas with features in progressive brain tumor. Lipoma HMGIC fusion partner-like 3 (LHFPL3) is reported to be highly expressed in malignant glioma, but the relationship and mechanism between LHFPL3 and tumor is inexplicit. The present study aimed to screen the miRNAs targeting LHFPL3 and verify the pathogenesis and development of gliomas. Bioinformatics software predicted that miR-218-5p and miR-138-5p can specifically bind to *LHFPL3* mRNA. And the expression of miR-218-5p and miR-138-5p was down-regulated in glioma cell lines and glioma tissues from the patients compared with the normal cells. While dual luciferase activity experiment confirmed, only miR-218-5p can directly bind to LHFPL3. After miR-218-5p transfection of U251 and U87 cells, cytological examinations found a reduction in cell activity, proliferation and invasive ability. Further study showed that miR-218-5p transfection could inhibit epithelial–mesenchymal transitions (EMT). Therefore, miR-218-5p targeting *LHFPL3* mRNA plays significant roles in preventing the invasiveness of glioma cells. The present study also revealed a novel mechanism for miRNA–LHFPL3 interaction in glioma cells, which may be potential targets for developing therapies in treating glioma.

## Introduction

Gliomas are primary brain tumors with histological features of glial cells [1,2]. According to the 2007 World Health Organization (WHO) system, gliomas are classified into four subtypes: grade III anaplastic astrocytoma, anaplastic oligodendroglioma, anaplastic oligoastrocytoma, and grade IV glioblastoma (GBM). Several molecular diagnostic tests are available on certain glioma specimens with the results telling prognostic or predictive implications such as 1p/19q deletion, *MGMT* methylation, and immunohistochemical staining for IDH1 and IDH2 mutation [3,4]. Despite these clinical applications, survival rate of gliomas has not significantly improved over the last decades. Therefore, in order to improve clinical outcomes, new strategies about treatment and prognosis are urgently needed to improve the current standard therapies.

Development of high-grade gliomas involves multiple tumorigenic events, including cell cycle control loss, dysregulation of apoptosis, growth factor overexpression, and angiogenesis [5]. Epithelial–mesenchymal transition (EMT) is a reversible biological process that occurs in epithelial cells [6,7]. Several EMT-inducing factors and signal pathway are discussed in gliomas such as Vimentin, Snail, and N-Cadherin [7,8]. It is reported that loss of E-cadherin function or expression is related to cancer progression and metastasis. Down-regulation of E-cadherin decreases the strength of cellular adhesion and enhance cellular motility. Increasing evidence miRNAs are highly evolved in tumor cell EMT [6,9].

miRNAs play important roles in the regulation of post-transcriptional gene expression, they are non-protein encoding RNAs and consist of 18–25 nts [10]. Increasing kinds of differentiated expressed miRNAs in gliomas have been identified by high-throughput profiling methods. Lipoma HMGIC fusion partner-like 3 (LHFPL3) is a novel found protein that might be characteristic of primary GBM [11,12]. LHFPL3 was altered in 33.3% of enrolled patients, predominantly in grade IV GBM samples in the present study. It was detected in significantly higher percentage in samples with high level of total genomic instability.

LHFPL3 may play a role in migration and invasion of GBM and the interaction between miRNAs and *LHFPL3* mRNA may participate in the EMT of glioma cells.

Here, in the present study, we found expression level of miR-218-5p was lower in patients glioma tissues compared with the level of normal brain tissues. This suggested miR-218-5p may play an important role in glioma. And, further study showed that miR-218-5p can directly bind to LHFPL3. Therefore, we further investigate the function of miR-218-5p by targeting LHFPL3 in glioma. Our study revealed, LHFPL3 is a novel target of miR-218-5p. The present results suggest an association between miR-218-5p-mediated down-regulation of glioma cell proliferation and the inactivation of EMT signaling related elements, and understanding the role of miR-218-5p may provide important insights into the treatment of gliomas or as a potential therapeutic candidate for miRNA replacement therapy [13]. Besides, the development of LHFPL3 as a biomarker for glioma is extremely promising.

## Materials and methods

### Clinical samples

Human glioma tumor tissue samples were obtained after patients received surgical resections from the People’s Hospital of Zhengzhou University (Zhengzhou, People’s Republic of China). The present study was approved by the ethics committee of the Ethics Committee of the People’s Hospital of Zhengzhou University, informed consent was obtained from every enrolled patient.

### Cell lines and transfection

Human brain normal glial cells (HEB), glioma cell lines U251, U87, T98-G, A172 were purchased from cell bank of Shanghai Institute for Biological Sciences. Cells were grown in DMEM medium supplemented with 10% FBS, 1% penicillin/streptomycin in an atmosphere at 37°C with 5% CO_2_. About 1 × 10^5^ U87 and U251 cells were seeded in six-well plates and transfected with miR-218-5p, miR-138-5p or Negative mimics using Lipofectamine 2000 (Invitrogen Life Technologies) following the manufacturer’s instructions. After 24 h, cells were placed in complete medium and maintained at 37°C in a 5% CO_2_ atmosphere.

### RNA extraction and reverse transcription quantitative PCR

Total RNA was extracted from the cultured cells and fresh glioma tissues using TRIzol reagent (Invitrogen Life Technologies). Total miRNAs were extracted with miRVana (Ambion, Austin, TX, U.S.A.). The expression level of miR-218-5p and miR-138-5p was quantitated using a miRNA specific TaqMan miRNA Assay kit and specifically designed primers (Applied Biosystems, Foster City, CA, U.S.A.).The expression levels of miR-218-5p, miR-138-5p, U6, GAPDH, and LHFPL3 were examined by quantitative PCR (qPCR) with a SYBR Green PCR Master Mix kit (Applied Biosystems, USA) in conjunction with an ABI-Prism 7300 System. The primer sequences were as follows: GAPDH, forward: 5′- TGTTCGTCATGGGTGTGAAC-3′; and reverse: 5′- ATGGCATGGACTGTGGTCAT-3′; U6, forward: 5′-CTCGCTTCGGCAGCACA -3′; and reverse: 5′-AACGCTTCACGAATTTGCGT -3′; LHFPL3, forward: 5′-ACCAACTATGTGCGGAACTCG -3′; and reverse: 5′-TCCACGCCGTCGCCTAT -3′. U6 and GAPDH were used as internal controls.

### Luciferase reporter assay

The LHFPL3 3′-UTR sequence was predicted to interact with miR-218-5p and miR-138-5p, LHFPL3 with a mutated sequence containing the predicted target sites were synthesized and cloned into the XhoI and NotI sites of a psiCHECK-2 control vector (Promega, U.S.A.). These constructs were termed psiCHECK-2-LHFPL-3′UTR-WT and psiCHECK-2-LHFPL-3′UTR-Mut. In the reporter assay experiment, the U87 cells were plated onto 24-well plates and transfected with psiCHECK-2-LHFPL-3′UTR-WT or psiCHECK-2-LHFPL-3′UTR-Mut, and miR-218-5p mimics, miR-138-5p mimics or Negative mimics using FuGENEHD (Promega, U.S.A.). After transfection for 48 h, the cells were harvested and assayed on the Dual-Luciferase Reporter Assay system (Promega) according to the manufacturer’s instructions. Transfection was repeated in triplicate in three independent experiments.

### Western blotting

Cell samples were lysed in RIPA buffer (Beyotime, Co., Ltd., People’s Republic of China) to obtain total cell lysates. Protein concentrations were determined using the bicinchoninic acid (BCA) protein assay kit (Thermo Fisher Scientific, Co., Ltd., U.S.A.). Protein (40 μg) from each sample was taken out to perform SDS/PAGE, and then transferred to PVDF membranes (Merck Millipore, Darmstadt, Germany) and further incubated with primary polycloncal antibodies: LHFPL3 (1:5000), E-cadherin (1:1000), N-cadherin (1:1500), vimentin (1:1000), snail (1:2000), followed by incubation with horseradish peroxidase conjugated monoclonal HRP goat anti-rabbit (BOSTER, No.BA1054, 1:20,000) and HRP Goat anti-Mouse IgG (BOSTER, No.BA1051,1:20,000) antibodies. The membranes were stripped and reprobed with a primary monoclonal mouse anti-rabbit antibody against GAPDH (1:1000 dillution; Bioworld, Nanjing, People’s Republic of China). GAPDH expression was used as a standard for the normalization of the measurement.

### Proliferation assays

According to the manufacturer’s protocol, the proliferative ability of glioma cells was measured in 4 days using the cell counting kit–8 (CCK–8) at 24, 48, and 72 h after miRNA transfection. CCK–8 solution (10 µl) was added to each well, incubated at 37˚C for 4 h in 5% CO_2_, the absorbance of each well was detected using a microplate reader (Multiskan Spectrum; Thermo Fisher Scientific, Inc., Waltham, MA, U.S.A.) at a wavelength of 450 nm.

5-ethynyl-29-deoxyuridine (EdU) incorporation assay was also implied for proliferative ability assessment. Cells were cultured in confocal dishes at a density of 1 × 10^5^ cells per dish for 24 h at 37°C. After transfected with 50 nM of miR-218-5p mimics or control for 48 h, 50 µM of EdU was added to each dish and cells were cultured for an additional 2 h at 37°C. The cells were fixed with 4% formaldehyde for 15 min at room temperature and treated with 0.5% Triton X-100 for 20 min at room temperature for permeabilization. After washing with PBS for three times, 100 µl of 1× Apollo^®^ reaction cocktail was added to each well and the cells were incubated for 30 min at room temperature. Then, the DNA contents of each well of cells were stained with 100 µl of Hoechst 33342 for 30 min and visualized under a fluorescence microscope (Olympus Corporation, Tokyo, Japan). The EdU incorporation rate was presented by the ratio of EdU-positive cells to total Hoechst 33342-positive cells.

### Transwell assay

Cell invasion ability was determined by Transwell assay. MiR-218-5p mimics were transfected into the cells according to the previously mentioned protocol. After incubation of glioma cells for 48 h, 3 × 10^4^ cells were transferred to the top of the Matrigel-coated invasion chambers (BD Biosciences, San Jose, CA, U.S.A.) in a serum-free Dulbecco’s modified Eagle’s medium (DMEM). DMEM containing 10% FBS was added to the lower chamber. After 24 h of cultivation, the non-invading cells were removed, and the invading cells were fixed using 95% ethanol, stained with 0.1% Crystal Violet and images were captured under a ×100 magnification. The tests were repeated in three independent experiments.

### Cell apoptosis assay

Cell apoptosis was detected by the flow cytometric analysis of Annexin V staining. The transfected cells were collected and washed with cold PBS twice. Cells were then resuspended in 1× binding buffer (BD Pharmingen) at a concentration of 1 × 10^6^ cells/ml. Solution (1 × 10^5^ cells (100 μl)) was transferred to a 5-ml culture tube. Phycoerythrin Annexin V (5 μl) and 5 ml propidium iodide (PI; BD Pharmingen) were added to the samples, and then incubated for 15 min at room temperature in the darkness. About 400 ml 1× binding buffer was added to each tube and the sample was analyzed by flow cytometry (FACSCanto II, BD Biosciences) immediately. FlowJo software was used for date analyze. Repeated experiments were performed in triplicate.

### Immunofluorescence assay

Expression of LHFPL3 protein level was also evaluated by immunofluorescence assay. U87 and U251 cell lines were transfected with miR-218-5p mimics and control plasmids for 48 h. The cells were then incubated in 4% paraformaldehyde, and fixed in ice-coldmethanol and blocked with 1% BSA, and bound with LHFPL3 (1:50) and actin (1:400) antibodies. The cells were incubated with anti-rabbit IgG (1:1000), anti-mouse IgG (1:1000) and Hoechst 33342 (Life Technologies Corp., NY). The cells were captured on a Nikon Eclipse Ti-U fluorescence microscope (Nikon Instruments, Inc.) equipped with a SPOT RTTM digital camera.

### Statistical analysis

Experimental data are displayed as mean ± S.D. All analyses were performed using a two-tailed Student’s *t*-test performed on SPSS software, version 12.0 (SPSS, Inc., Chicago, IL, U.S.A.), and a *P*-value <0.05 was considered to indicate statistical significance difference.

## Result

### MiR-218-5p and miR-138-5p were down-regulated in glioma cell lines and tissues

**Figure 1:**
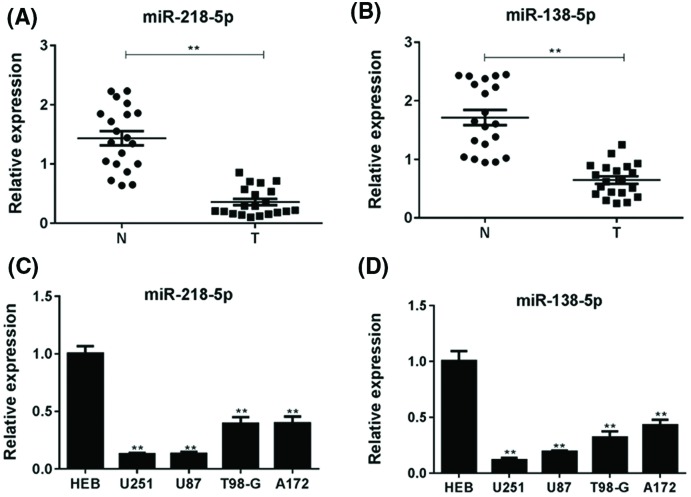
MiR-218-5p and miR-138-5p were down-regulated in tissues and glioma cells The relative expression level of miR-218-5p (**A**) and miR-138-5p (**B**) in glioma samples and non-tumor brain tissues by real-time qRT-PCR. **P<0.01 compared with non-tumor brain tissues. The expression of miR-218-5p (**C**) and miR-138-5p (**D**) in various glioma cell lines (U251, U87, T98-G, A172) and a normal glial cell line (HEB) by real-time qRT-PCR. **P<0.01 compared with HEB cells.

To understand the relationship between miRNA and glioma cell malignancy, the expression of miR-218-5p and miR-138-5p in the human glioma tissues and normal tissues was detected by qPCR analysis. We found that both miR-218-5p and miR-138-5p had a lower expression in glioma tissues than normal tissues (Figure 1A,B). Moreover, the expression of miR-218-5p and miR-138-5p was tested in U251, U87, T98-G, A172 cell lines and HEB cells. MiR-218-5p and miR-138-5p expression in glioma cell lines, especially in U251 and U87, were significantly decreased (*P*<0.05) compared with HEB cells (Figure 1C,D). These results suggested that miR-218-5p and miR-138-5p were down-regulated in glioma cell lines and glioma tissues. U251 and U87 cell lines were chosen for further *in vitro* experiments.

### LHFPL3 is a direct target of miR-218-5p in glioma cell

**Figure 2:**
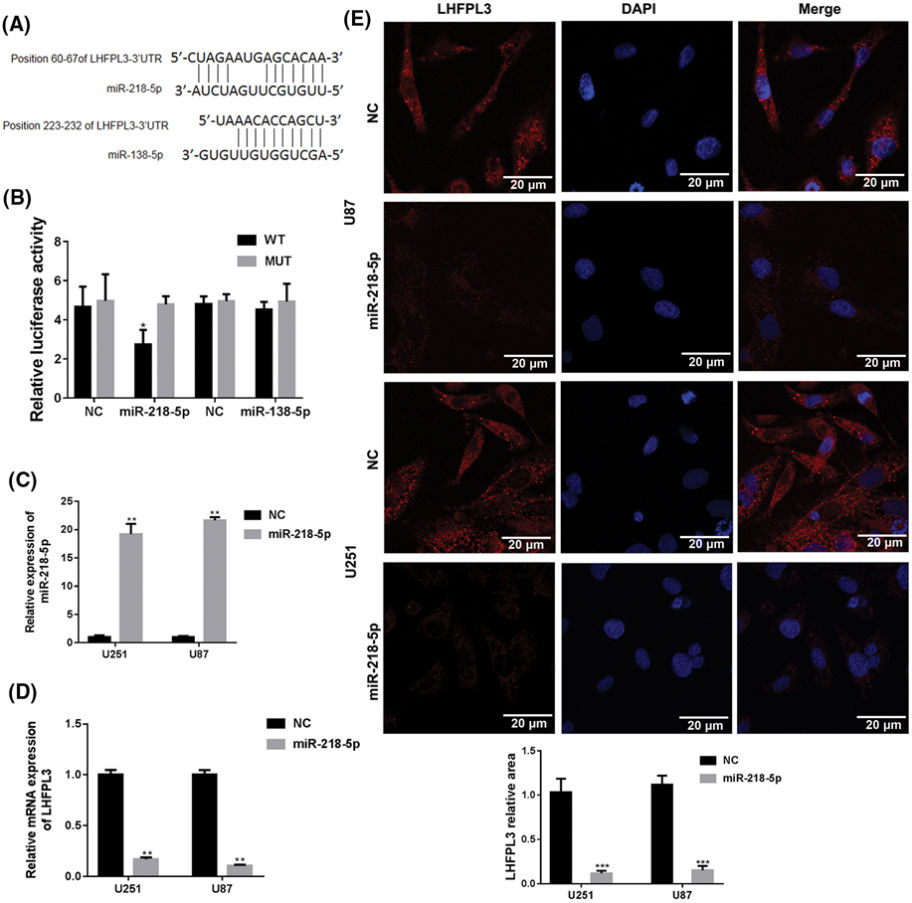
LHFPL3 was a direct target of miR-218-5p (**A**) Identification of target sites in the 3′-UTR of LHFPL3 was performed in TargetScan. (**B**) Luciferase assay was applied to test the target relationship between LHFPL3 mRNA and miRNAs in U87 cells. Luciferase activity was evaluated as described in materials and methods. Representative of at least three independent experiments. *P<0.05 compared with LHFPL3-WT group. (**C**) The expression levels of miR-218-5p and LHFPL3 (**D**) were detected by qRT-PCR after miR-218-5p mimics transfection both in U87 and U251 cells. **P<0.01 compared with NC group. (**E**) Immunofluoresence staining of LHFPL3 in glioma cells transfected with miR-218-5p mimics. The photographs were shown at ×120 original magnification. The scale bar is 20 um. ***P<0.001 compared with NC group.

Based on results of screening in online prediction tools, including miR and, TargetScan, and RNA hybrid, LHFPL3 was chosen as the candidate target gene of miR-218-5p and miR-138-5p (Figure 2A). Furthermore, luciferase reporter assays were taken to detect the binding ability of miR-218-5p and miR-138-5p to the 3′-UTR of LHFPL3. As shown in Figure 2B, luciferase activity of LHFPL3-WT was significantly inhibited by co-transfection with miR-218-5p mimics than NC (*P*<0.05) but LHFPL3-MUT was not changed. However, the luciferase activity of LHFPL3-WT was not changed by miR-138-5p mimics obviously (Figure 2B). The result demonstrated that LHFPL3 is a direct target of miR-218-5p rather than miR-138-5p. Therefore, we continued to study the mechanism between LHFPL3 and miR-218-5p in giloma cells.

To detect the effect of miR-218-5p on LHFPL3 in glioma cells, we transfected miR-218-5p mimics in U251 and U87 cells to up-regulate the expression of miR-218-5p. After treated with miR-218 mimics for 24 h, the miR-218-5p expression was significantly up-regulated (Figure 2C) and a decreased expression of LHFPL3 was observed through qPCR assays (Figure 2D). Furthermore, through immunofluorescence staining of LHFPL3 in U251 and U87 cells transfected with miR-218-5p or NC *in situ*, the density of LHFPL3 was weaker in miR-218-5p than NC group (Figure 2E). These results indicated that the elevated miR-218-5p could inhibit LHFPL3 expression.

### MiR-218-5p inhibited cell proliferation

**Figure 3:**
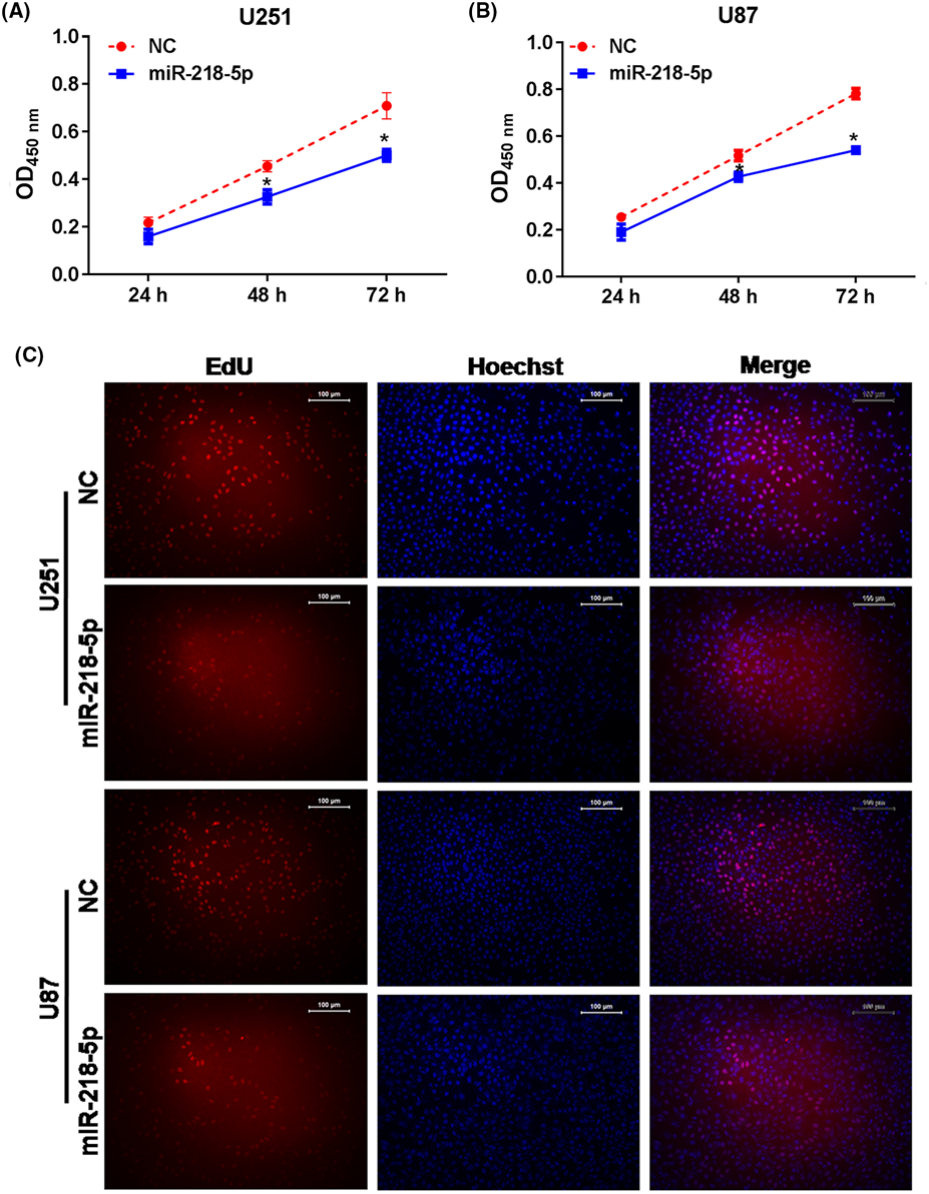
The effects of miRNA-218-5p mimics on cell proliferation ability *in vitro* MiR-218-5p mimics can inhibit cell proliferation in U251 (**A**) and U87 cells (**B**) by CCK-8 assay, cell density significantly decreased after miRNA-218-5p mimics transfection at 24, 48, and 72 h. *P<0.05 compared with NC group. (**C**) The percentage of EdU-positive cells in U87 and U251 cells was observed after treated with *miRNA-218-5p* mimics for 48 h. The photographs were shown at ×100 original magnification.

To explore the effect of miR-218-5p on glioma cell proliferation, CCK-8 assays were performed after transfected with miR-218-5p mimics. We found a decreased cell proliferation rate in the miR-218-5p group compared with NC group after transfection for 48 and 72 h both in U251 and U87 cells significantly (Figure 3A,B). Besides, EdU assay was taken to further confirm the proliferation capacity of U251 and U87 after transfected with miR-218-5p mimics for 48 h. As shown in Figure 3C, overexpression of miR-218-5p could reduce the viability of U251 and U87 cells.

### MiR-218-5p induced cell cycle arrest and apoptosis

**Figure 4:**
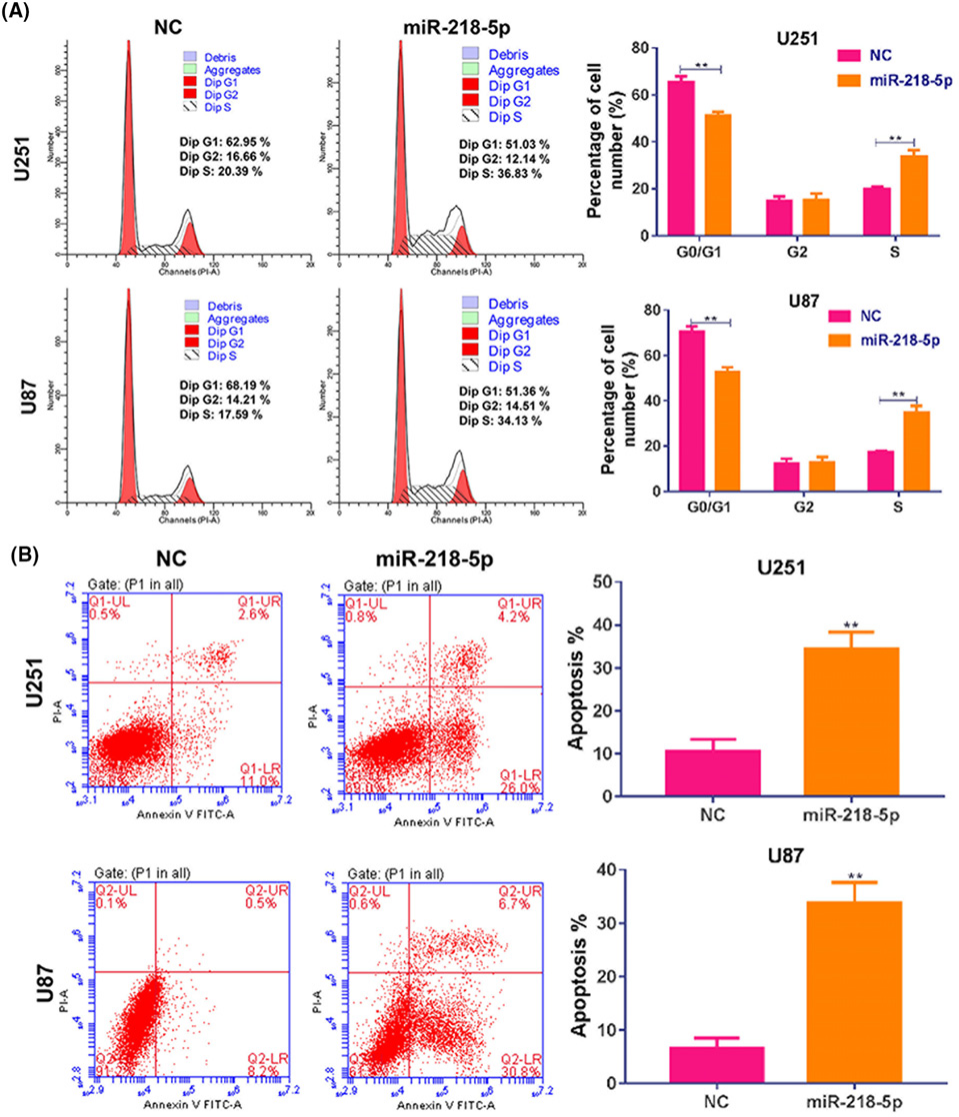
MiR-218-5p mimics disturbed distribution cell cycle and induced apoptosis (**A**) The status of cell phases was detected by flow cytometry after miR-218-5p mimics transfection for 48 h in U251 and U87 cells. (**B**) The percentage of apoptotic cells was detected by flow cytometry after miR-218-5p mimics transfection for 48 h in U251 and U87 cells. **P<0.01 compared with NC group.

Furthermore, cell cycle and apoptosis were examined by flow cytometry. Compared with NC group, miR-218-5p group showed that the proportion of G_0_/G_1_-phase cells decreased, while the proportion of S-phase increased significantly, indicating that overexpression of miR-218-5p induced a S-phase arrest in glioma cells (Figure 4A). The proportion of apoptotic cells induced by transfection of miR-218-5p was also observed in U251 and U87 cells in contrast of control group (Figure 4B), indicating that miR-218-5p overexpression could significantly induce the apoptosis of glioma cells.

### MiR-218-5p inhibited cell invasion and EMT signal pathway

**Figure 5:**
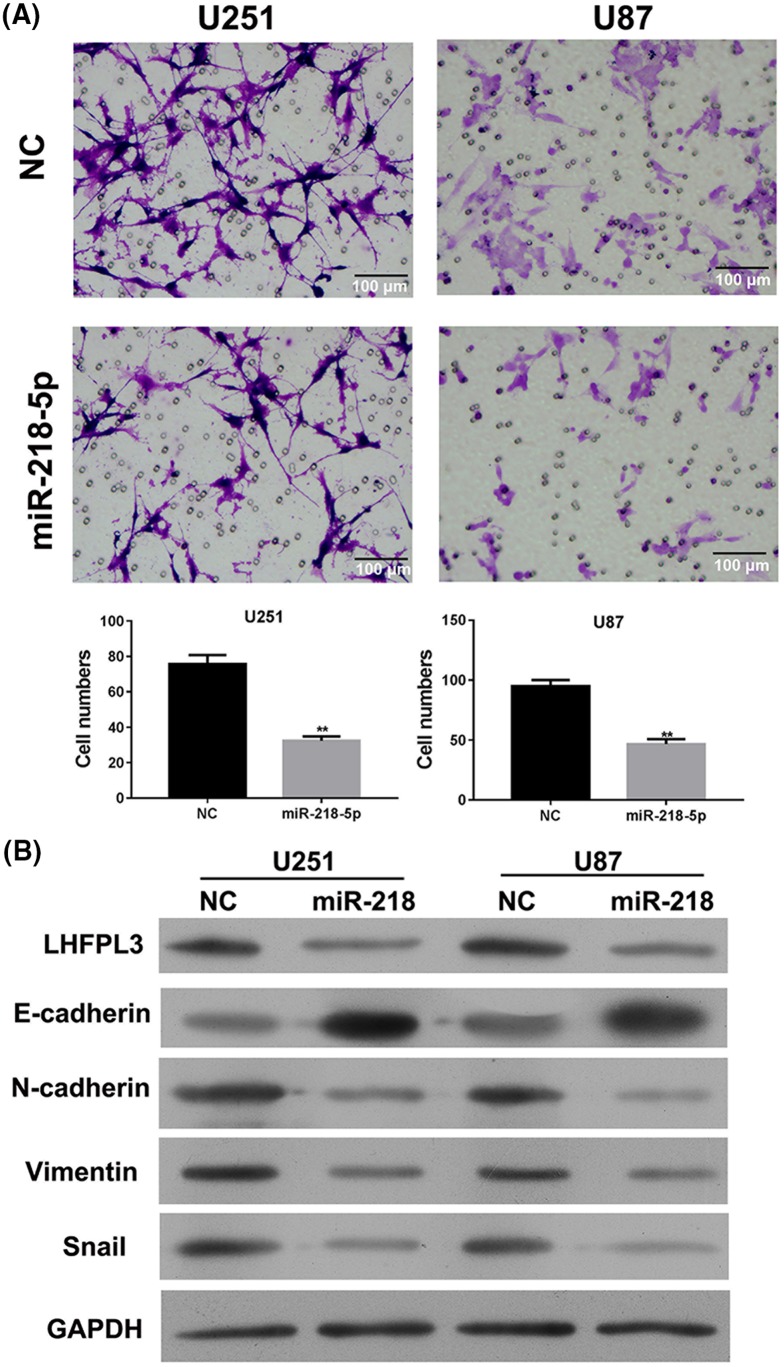
MiR-218-5p regulated the invasive ability and the protein levels of EMT markers in U87 and U251 cells (**A**) Representative micrographs of migrated U251 and U87 cells were observed after miR-218-5p mimics transfection by Transwell migration assays. The scale bar is 100 um. **P<0.01 compared with NC group. (**B**) The protein expression of LHFPL3 and EMT markers (E-Cadherin, N-Cadherin, Vimentin, and Snail) in U251 cells (left) and U87 cells (right) was carried out after transfected with miR-218-5p mimics by Western blot assay. All experiments performed in triplicate.

To study the effect of miR-218-5p on cell invasion, Transwell invasion assay was taken 48 h after miR-218-5p was transfected in U251 and U87 cells. As shown in Figure 5A, the number of U251 and U87 cells invading through the Matrigel of invasion chamber was significantly reduced in miR-218-5p group, indicating that overexpression of miR-218-5p could inhibit glioma cells invasion (Figure 5A). In addition, to study the effect of miR-218-5p on EMT signal pathway, we determined the protein levels of EMT related proteins, including E-Cadherin, N-Cadherin, Vimentin, and Snail by Western blot. LHFPL3 level was down-regulated by miR-218-5p transfection both in U251 and U87 cells. E-cadherin levels were increased while N-cadherin, Vimentin, and Snail levels were decreased in U251 and U87 cells after transfected with miR-218-5p mimics, compared with control cells (Figure 5B). These results indicate that miR-218-5p could reduce the expression of LHFPL3 and inhibit activation of EMT signal pathway simultaneously.

### MiR-218-5p inhibitor reversed the function of LHFPL3 siRNA inhibited cell proliferation

**Figure 6:**
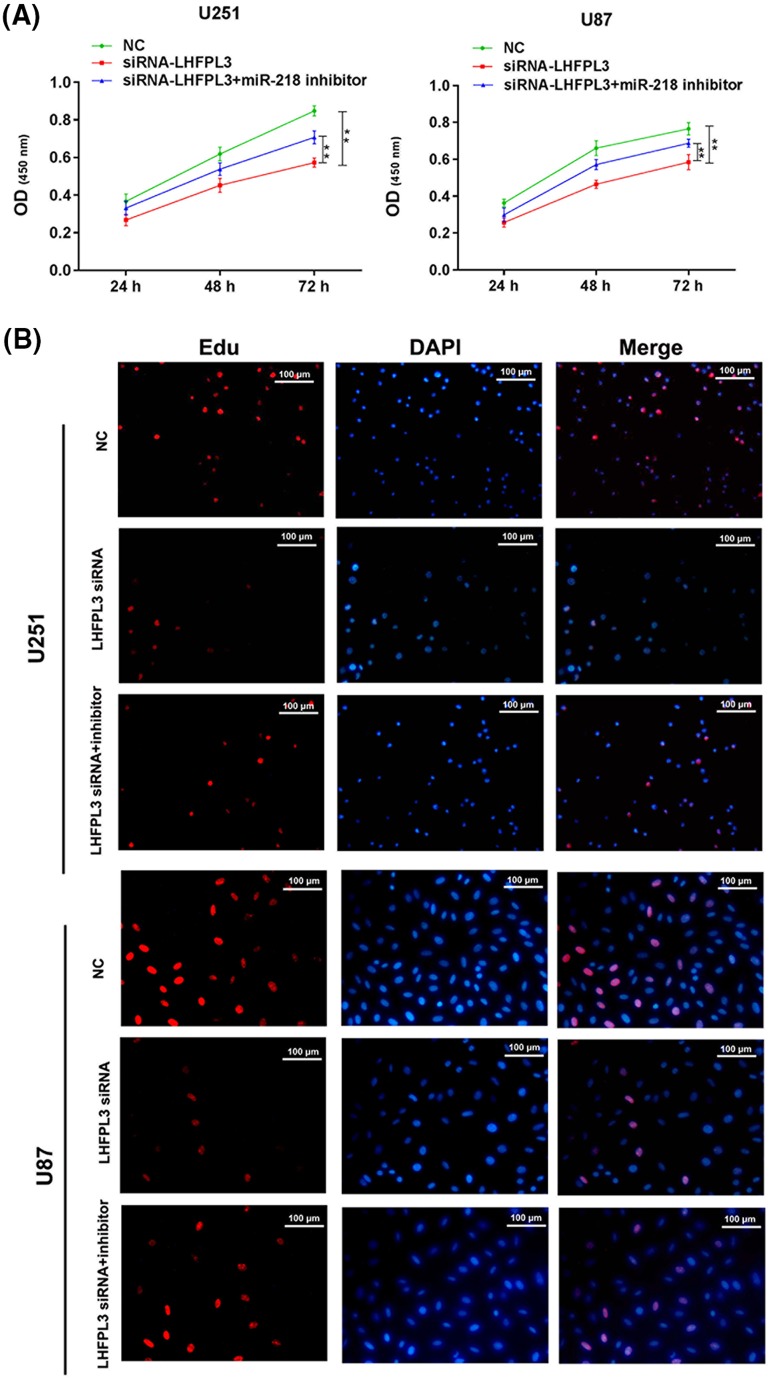
MiR-218-5p inhibitor partly reversed the suppressive proliferation of LHFPL3 siRNA Proliferation ability was detected by CCK-8 assay (**A**) and Edu experiment after LHFPL3 siRNA or LHFPL3 siRNA and miR-218-5p inhibitor transfection. *P<0.05, LHFPL3 siRNA compared with miR-218-5p inhibitor + LHFPL3 siRNA. (**B**). The scale bar is 100 μm.

To further investigate whether miR-218-5p exerted its function by targeting LHFPL3, we co-transfected miR-218-5p inhibitor and LHFPL3 siRNA in U251 and U87 cells and, then CCK-8 assay (Figure 6A) and Edu assay (Figure 6B) were conducted to test the proliferation ability. The results showed, LHFPL3 siRNA can inhibit proliferation, similar to the function of miR-218-5p. Besides, miR-218-5p inhibitor can partly reverse the function of LHFPL3 siRNA, suggesting miR-218-5p may exact its function in proliferation by targeting LHFPL3.

### MiR-218-5p inhibitor partly reversed the function of LHFPL3 siRNA in cell cycle and apoptosis

**Figure 7:**
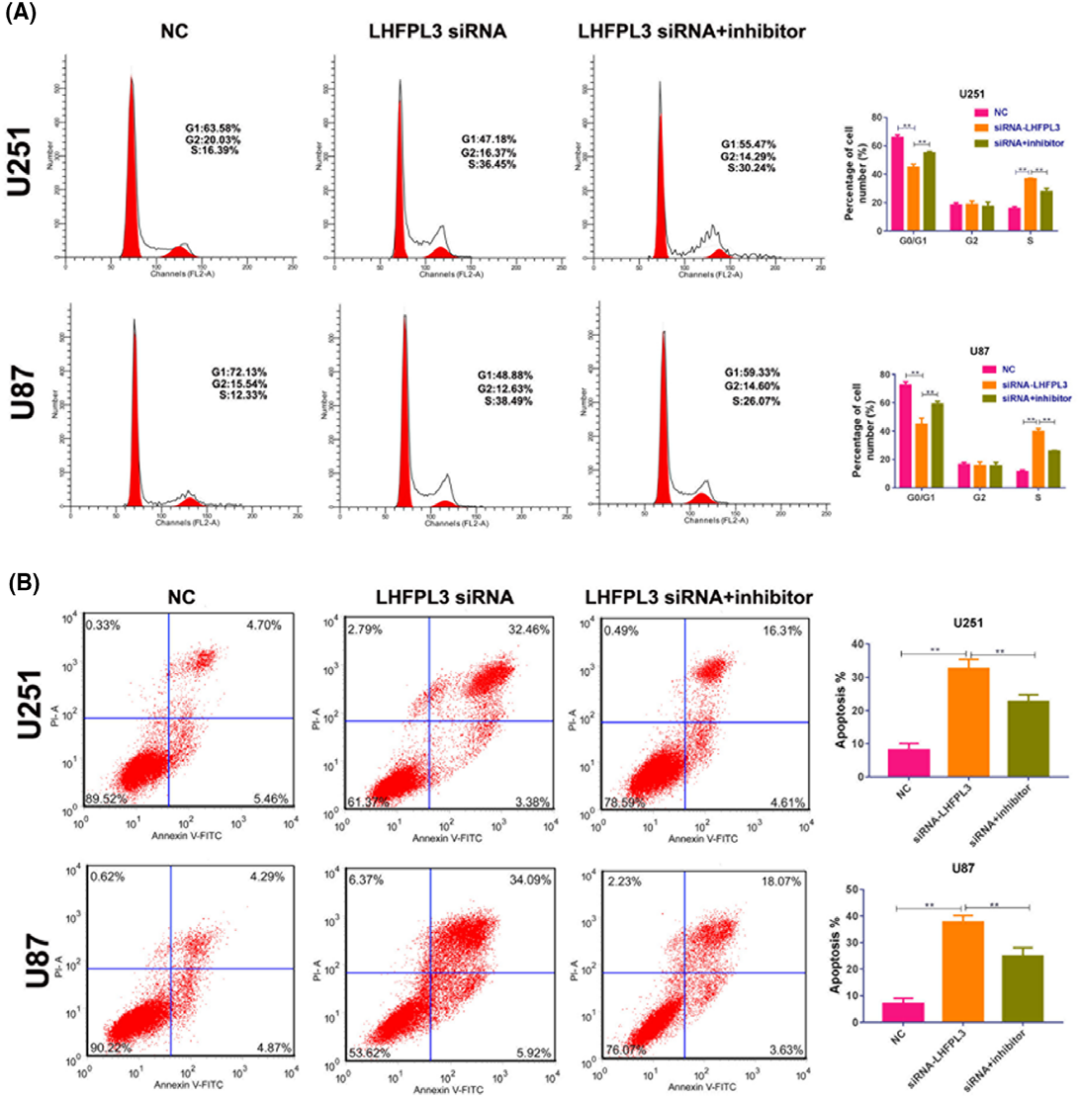
MiR-218-5p inhibitor partly restored the function of LHFPL3 siRNA on cell cycle and apoptosis Cell cycle distribution and apoptosis (**A,B**) were analyzed by flow cytometry after LHFPL3 siRNA or LHFPL3 siRNA and miR-218-5p inhibitor transfection. **P<0.01.

After miR-218-5p inhibitor and LHFPL3 siRNA were co-transfected, cell cycle and apoptosis were also analyzed. Our data showed, LHFPL3 siRNA can induce cell cycle arrest (Figure 7A) and apoptosis (Figure 7B), similar to miR-218-5p. And, miR-218-5p inhibitor can partly reverse the function of LHFPL3 siRNA. These results suggested miR-218-5p may play its function in cell cycle and apoptosis by targeting LHFPL3.

### MiR-218-5p inhibitor partly reversed the function of LHFPL3 siRNA in EMT

**Figure 8:**
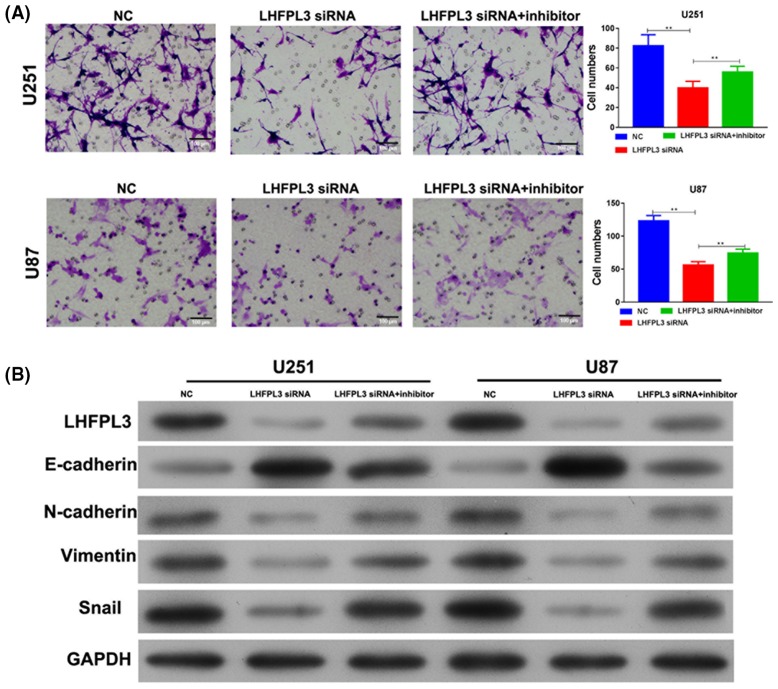
MiR-218-5p inhibitor partly reversed the effect of LHFPL3 siRNA on invasion (**A**) Invasion ability was analyzed by Transwell assay after LHFPL3 siRNA or LHFPL3 siRNA and miR-218-5p inhibitor transfection. **P<0.01. (**B**) Western blot was used to analyze the expression level of EMT markers (E-Cadherin, N-Cadherin, Vimentin, and Snail).

In addition, Transwell assay showed, LHFPL3 siRNA can inhibit invasion ability, similar to miR-218-5p (Figure 8A). Furthermore, miR-218-5p inhibitor can restore the effect of LHFPL3 siRNA (Figure 8A). Western blot showed, LHFPL3 siRNA induced E-cadherin expression and reduced the expression of N-cadherin, Vimentin, and Snail. Conversely, miR-218-5p inhibitor can restore the action of LHFPL3 siRNA in these EMT markers (Figure 8B). Taken together, our results proved miR-218-5p may exert its function by targeting LHFPL3.

## Discussion

The most important finding of the present study is that miR-218-5p expression was significantly differentiated in glioma cells and glioma tissues, compared with the expression level in primary normal human astrocytes (HEB), and miR-218-5p could regulate the post-transcriptional status of LHFPL3 directly. We also showed that miR-218-5p inhibits proliferation, apoptosis, invasive ability of glioma cells.

GBM multiforme is the most common lethal cancer in brain tumor, which is incurable with a median survival time of only 15 months [14]. There are many efforts in identifying new targets for GBM regarding to diagnostics and therapeutics [12,15,16]. Under the trend of precision medicine and the development of high throughput technology, recent studies have revealed a lot of biomarkers in glioma [13,17]. One of the most interesting finding focus on miRNAs due to their regulatory ability in both normal development and in pathological conditions in cancer [18,19]. miRNAs have a wide range of targets, which play critical to cancer progression, including apoptosis [20], proliferation [21], cell death, angiogenesis [22], metastasis [23], and drug resistance [24]. After screening of numbers of miRNAs with potential applications such as miR-203 [25], miR-200 [26], miR-10b, miR-21 [27], and miR-16 [28] which were located on separated miRNA clusters, the targets and mechanism of miRNAs become another critical question which is urgent to be answered.

Alterations of lipoma HMGIC fusion partner (LHFPL3) were found to be more frequent in grade IV GBM, the survival rate of patients with mutations of LHFPL3 was significantly worse than the survival of patients without these alterations [12]. This finding show the perspective that LHFPL3 may be applied in glioma diagnosis, classification or prognosis [29–31]. The luciferase activity assay analysis confirmed that the down-regulation of LHFPL3 was mediated by miR-218-5p through its binding to the 3′UTR of LHFPL3 mRNA. Our results showed that miR-218-5p significantly decrease the expression level of intracellular LHFPL3 by post-transcriptional regulation, and inhibited cell function in glioma. In the present study, we first found that LHFPL3 was a theoretical target of miR-218-5p through bioinformatic analysis, and next confirmed that LHFPL3 was a target of miR-218-5p.

EMT plays an important role in tumor development [6,9]. Snail is a powerful repressor of E-cadherin transcription and an important regulator in EMT. EMT ultimately leads to the formation of a mesenchymal phenotype, causing increased cell motility and resistance to treatments [16,32]. Previous studies found that Snail was regulated at both the transcriptional and post-transcriptional levels [33,34]. Signaling molecules such as E-Cadherin, N-Cadherin, Vimentin was regulated in different levels in EMT signal pathway post-transcriptionally. In addition, E-cadherin, the downstream target of Snail, was noticeably regulated by the overexpression of miR-218-5p.

In summary, the present study explained, for the first time, an important role between miR-218-5p and its target LHFPL3. The interaction mechanism of these two elements will elucidate the precise role of miR-218-5p and LHFPL3 in glioma progression, which will not only increase our knowledge of the pathogenesis of glioma but also enlightening in future novel therapeutic strategies. Regarding the result of miR-218-5p and EMT related proteins, more comprehensive mechanism links LHFPL3 and EMT will be addressed. Moreover, targeting the interaction between miR-218-5p and LHFPL3 or restoring miR-218-5p expression may be new therapeutic methods to treat glioma patients in the future.

## Abbreviations

CCK-8: cell counting kit–8
DMEM: Dulbecco’s modified Eagle’s medium
EdU: 5-ethynyl-29-deoxyuridine
EMT: epithelial–mesenchymal transition
GBM: glioblastoma
HEB: human brain normal glial cell
HRP: Horseradish Peroxidase
IDH: Isocitrate dehydrogenases
LHFPL3: lipoma HMGIC fusion partner-like 3
MGMT: O-6-methylguanine-DNA methyltransferase
NC: Negative Control
